# Local chemerin levels are positively associated with DSS-induced colitis but constitutive loss of CMKLR1 does not protect against development of colitis

**DOI:** 10.14814/phy2.12497

**Published:** 2015-08-11

**Authors:** Helen J Dranse, Jillian L Rourke, Andrew W Stadnyk, Christopher J Sinal

**Affiliations:** 1 Department of Pharmacology, Dalhousie UniversityHalifax, Nova Scotia, Canada; 2 Department of Microbiology and Immunology, Dalhousie UniversityHalifax, Nova Scotia, Canada; 3 Department of Pediatrics, Dalhousie UniversityHalifax, Nova Scotia, Canada

**Keywords:** Adipokine, chemerin, CMKLR1, colitis, inflammatory bowel disease

## Abstract

Inflammatory bowel disease (IBD) is a family of disorders including ulcerative colitis and Crohn’s disease that are characterized by chronic and relapsing intestinal inflammation. Increased production of proinflammatory mediators, possibly combined with low expression of anti-inflammatory mediators, is thought to promote the development and progression of IBD. In the current study, we demonstrate that expression, secretion, and processing of chemerin, a potent chemoattractant for cells expressing chemokine-like receptor 1 (CMKLR1), increased in the cecum and colon along a gradient positively associated with the severity of inflammation in dextran sodium sulfate (DSS)-induced colitis. We also show that levels of circulating bioactive chemerin increased following DSS treatment. At both 6–8 and 14–16 weeks of age, CMKLR1 knockout mice developed signs of clinical illness more slowly than wild type and had changes in circulating cytokine levels, increased spleen weight, and increased local chemerin secretion following DSS treatment. However, knockout mice ultimately developed similar levels of clinical illness and local inflammation as wild type. Finally, contrary to previous reports, intraperitoneal injection of bioactive chemerin had no effect on the severity of DSS-induced colitis. This suggests that local chemerin levels have a greater impact than circulating levels in the pathogenesis of colitis. Considered altogether, bioactive chemerin represents a novel biomarker for IBD severity, although strategies to modulate endogenous chemerin signaling other than chronic CMKLR1 loss are necessary in order to exploit chemerin as a therapeutic target for the treatment of IBD.

## Introduction

Inflammatory bowel disease (IBD) is a family of disorders characterized by chronic relapsing inflammation and mucosal damage in the gastrointestinal (GI) tract. IBD is believed to develop in response to a combination of genetic and environmental factors with the two main forms, Crohn’s disease (CD) and ulcerative colitis (UC), having distinct pathologies (Hanauer 2006; Kaser et al. 2010; Ford et al. 2013; Frolkis et al. 2013). In both disorders, inflammation involves diffuse leukocyte infiltration and increased levels of proinflammatory cytokines, resulting in damage and disruption of the epithelium and the formation of ulcers, crypt abscesses, and in the case of CD, fistulas and strictures (Hanauer 2006; Kaser et al. 2010). IBD is associated with abdominal pain, diarrhea, rectal bleeding, and a significantly compromised quality of life (Ford et al. 2013). Common treatment approaches include anti-inflammatory (e.g., 5-aminosalicylates, glucocorticosteroids), immunosuppressant (e.g., thiopurines), and biologic (e.g., anti-TNF) agents that modulate the function of chemokine and cytokine networks that mediate intestinal inflammation (Ford et al. 2013; Bernstein 2015). However, available treatments are known to produce undesirable effects (e.g., glucocorticoid-stimulated bone loss) and often suffer from high relapse rates (Podolsky 2002; Ford et al. 2013; Bernstein 2015). Thus, current research continues to be focused on the signaling networks that underlie the development and progression of IBD in attempts to identify novel therapeutic targets.

Chemerin is an 18-kDa secreted protein that was first isolated from human inflammatory fluids and has since been positively associated with a number of diseases involving chronic inflammation (Wittamer et al. 2003). These include kidney disease, pancreatitis, pre-eclampsia, polycystic ovary syndrome, obesity, and liver disease (for review, see Rourke et al. 2013; Mariani and Roncucci 2015). Chemerin has also been characterized as an adipokine and secretion of the inactive precursor, prochemerin, from white adipose tissue, in addition to the liver, is thought to be the primary source of circulating chemerin (Goralski et al. 2007). Circulating or locally expressed prochemerin undergoes extracellular processing to generate a variety of bioactive isoforms (for review, see Rourke et al. 2013). Bioactive chemerin binds and activates two different G protein-coupled receptors – chemokine-like receptor 1 (CMKLR1) and G protein-coupled receptor 1 (GPR1) (Meder et al. 2003; Wittamer et al. 2003; Barnea et al. 2008; Rourke et al. 2013). Chemerin has also been shown to bind to C-C chemokine receptor-like 2 (CCRL2), which is believed to be a nonsignaling chemerin receptor (Zabel et al. 2008). All three receptors have been shown to play functional roles in mediating chemerin action in inflammation, immunity, and metabolism (Rourke et al. 2014; for review, see Rourke et al. 2013; Zabel et al. 2014). However, the function of chemerin as a potent chemoattractant for leukocytes that express CMKLR1, including macrophages, dendritic cells, and natural killer cells, has received particular attention (Wittamer et al. 2003; Zabel et al. 2005). Numerous in vitro and animal studies have demonstrated that chemerin/CMKLR1 signaling plays an essential role in the recruitment of CMKLR1-expressing cells to sites of localized inflammation or tissue damage (Rourke et al. 2013; Mariani and Roncucci 2015). Importantly, chemerin has been shown to play a role in the recruitment of macrophages to the developing fetal intestine (Maheshwari et al. 2009). With these biological activities in mind, recent studies have focused on whether chemerin plays an active role in IBD.

Clinical studies have demonstrated that circulating total chemerin levels are elevated in patients with both UC and CD and that chemerin expression is higher in colon biopsies from inflamed versus healthy regions in UC patients (Weigert et al. 2010; Lin et al. 2014). Furthermore, animal studies have shown that injection of bioactive chemerin exacerbates the severity of colitis (Lin et al. 2014). Given that IBD is characterized by increased infiltration of immune cells to the colon, we hypothesized that chemerin signaling plays a proinflammatory role in IBD and that a loss of CMKLR1 would protect against development of the disease. We demonstrate that endogenous chemerin expression, secretion, and bioactivity indeed increase with the onset of DSS-induced colitis. In addition, we directly investigate the role of CMKLR1 in an animal model of IBD. We show that while a loss of CMKLR1 does not protect against development of DSS-induced colitis, CMKLR1 KO mice have a slower onset of clinical illness and altered systemic inflammatory parameters.

## Methods

### Animals

C57BL/6 mice were obtained from the Jackson Laboratory (Bar Harbor, ME). CMKLR1 knockout mice were fully backcrossed onto the C57Bl/6 background and have been previously described (Ernst et al. 2012). All studies were performed with male animals 6–8 weeks of age unless otherwise indicated. For studies involving CMKLR1 heterozygous (HET) and knockout (KO) mice, breeding were between male and female mice heterozygous for CMKLR1 such that littermates of all three genotypes were available and analyzed. Mice were maintained under specific pathogen-free conditions, at 21°C in a 12 h dark and light cycle with free access to food and water. Animals were sacrificed using an overdose (90 mg/kg) of pentobarbital sodium injected intraperitoneally followed by exsanguination via cardiac puncture. All experimental protocols were approved by the Dalhousie University Committee on Laboratory Animals and in accordance with the Canadian Council on Animal Care guidelines.

### Dual-energy X-ray absorptiometry (DEXA)

Mice were anesthetized using isoflurane and body composition was assessed using a GE Lunar Piximus 2 densitometer and LUNAR PIXImus2 software (GE Medical Systems, Milwaukee, WI). The DEXA instrument was calibrated before each use and the head region of each mouse was excluded from analysis.

### DSS-induced colitis model

Experimental UC was induced as described previously (Jain et al. 2013). Briefly, facility drinking water was supplemented with 5% or 3.5% w/v DSS (36,000–50,000 MW; MP Biomedicals, Solon, OH) for 5 days ad libitum. On day 5, mice were returned to normal drinking water. Healthy control subjects received normal drinking water throughout the study. Water consumption levels were measured daily and no difference between cages was detected (data not shown). Body mass, stool consistency, and presence of blood in the stool (determined using FOB Test; Innovatek Medical Inc., Delta, BC) were monitored daily. Clinical scores were assigned based on a scoring system previously described (Stillie and Stadnyk 2009). Mice were sacrificed on day 6 (e.g., 1 day after removal of DSS) and the spleen was resected and weighed. The colon was excised, measured, flushed with PBS, and then divided longitudinally into pieces for further analysis as described below.

For experiments involving injection of chemerin, 500 ng of recombinant BSA-free mouse chemerin protein (aa17–156; R&D, Minneapolis, MN) or PBS (control) was injected intraperitoneally every other day starting on day 0. Recombinant chemerin contained <0.01 endotoxin units per 1 *μ*g protein as assessed by the manufacturer using the limulus amebocyte lysate test.

### Explant cultures

Adipose tissue was removed from excised colons and the colon was divided into five pieces, rinsed three times with PBS containing penicillin (100 U/mL) and streptomycin (100 *μ*g/mL), and placed in a 24-well plate (Costar, Corning, NY) containing 1 mL high glucose (4.5 g/L) DMEM with 10 mmol/L HEPES, 0.5% fetal bovine serum, 2 mmol/L l-glutamine, 50 *μ*mol/L 2-mercaptoethanol, penicillin, and streptomycin. Colon explants were cultured at 37°C with 5% CO_2_. After 24 h, explants were collected, pelleted by centrifugation, and the tissue pellet was weighed. The supernatant was stored at −80°C in aliquots for further use.

### Histology and immunohistochemistry

A longitudinal piece of colon was rolled using the “Swiss roll” method. Rolled colons were fixed in 10% neutral buffered formalin for 24 h and then transferred to 70% ethanol. Colons were then processed for paraffin embedding, 4 *μ*m sections were cut from the centre of each roll, and stained with hematoxylin and eosin. Sections were assessed for signs of inflammation by a blinded investigator based on parameters previously described (Stillie and Stadnyk 2009). Briefly, a score was assigned based on the presence of edema (0–1), ulceration (0–3), hyperplasia (0–3), crypt damage (0–5), and inflammatory infiltrate (0–5).

For immunohistochemistry, colon sections were deparaffinized, hydrated through a series of ethanol washes (100%, 95%, and 70%), and antigen retrieval performed via incubation with 20 *μ*g/mL proteinase K (Promega, Madison, WI) for 15 min. Slides were then incubated with 1:25 rat anti-mouse CMKLR1 (AbCam, Boston, MA) overnight at 4°C. The next day, colon sections were washed with PBST, incubated with 3% hydrogen peroxide for 10 min, and then incubated with biotinylated anti-rat secondary (BioLegend, San Diego, CA) for 1 h at room temperature. Slides were washed, incubated with avidin/biotin complex for 30 min (Vector Laboratories, Burlingame, CA) at room temperature, and developed with DAB staining (Vector Laboratories) for 3 min. Slides were then stained with hematoxylin, dehydrated, and mounted with Cytoseal60 (Fisher Scientific, Ottawa, ON). Images were taken using an Axioplan II microscope, Axiocam HRC color camera, and Zen 2012 Lite software (Zeiss, Toronto, ON). Representative images of three independent experiments are shown.

### RNA isolation and quantitative PCR (qPCR)

Immediately after sacrifice, GI tissues were excised and carefully cleared of any attached adipose tissue. White adipose tissue (WAT) samples were isolated from the mesenteric adipose depot. Tissues were cut into pieces and placed into TriZol (Ambion, Carlsbad, CA). Samples were homogenized using PowerGen700 (Fisher Scientific) and RNA was isolated according to manufacturer’s instructions until after the addition of 70% ethanol, at which point the sample was transferred to an RNAeasy column (Qiagen, Valencia, CA). RNA was further isolated according to manufacturer’s recommendations including the on-column DNA digestion step. RNA was quantified using FLUOstar Omega (BMG LabTech, Cary, NC). Approximately 2 *μ*g of RNA was transcribed to cDNA using EcoDry Premix Double-primed (Clontech, Mountain View, CA). cDNA was then diluted to 0.25 *μ*g/*μ*L and qPCR performed using 1 *μ*L of cDNA per reaction, with 0.5 *μ*mol/L primers, and FastStart SYBR green master mix (Roche, Laval, QC). qPCR was performed on LightCycler96 according to manufacturer’s recommendations and analyzed using LightCycler96 software (Roche). Relative gene expression was calculated using the ddCt method (Livak and Schmittgen 2001), where each sample was normalized to cyclophilin A as the reference gene. Primer sequences were as follows: Chemerin (NM_027851) – 5′-TACAGGTGGCTCTGGAGGAGTTC, 3′-CTTCTCCCGTTTGGTTTGATTG; CMKLR1 (NM_008153) – 5′-GCTTTGGCTACTTTGTGGACTT, 3′-CAGTGTTCACGGTCTTCTTCATCTTG; GPR1 (NM_146250) – 5′-ATTCAGCGCAGGCACCTTTC, 3′-CAAGCTGTCGTGGTGTTTGA; CCRL2 (NM_017466) – 5′-CTCTGCTTGTCCTCGTGCTT, 3′-GCCCACTGTTGTCCAGGTAG; and Cyclophilin A (NM_008907) – 5′-GAGCTGTTTGCAGACAAAGTTC, 3′-CCCTGGCACATGAATCCTGG.

### Blood collection

For experiments involving daily collection of serum, blood was collected from the saphenous vein of alert mice. For experiments involving serum collection at sacrifice, blood was collected via cardiac puncture after mice had been administered a lethal dose of sodium pentobarbital. For both, blood was allowed to clot at room temperature for 2 h, spun at 2000 *g* for 20 min, and then the serum collected and stored in aliquots at −80°C until further use.

### Analysis of cytokine and chemerin levels

Inflammatory cytokine levels (IL-6, IL-17a, IFN-*γ*, and TNF) were determined in colon explant supernatant (no dilution) and serum (1:4 dilution) using a Bio-plex multiplex assay according to manufacturer’s instructions (Bio-Rad, Hercules, CA) using a Bio-Plex 200 luminometer (Bio-Rad).

Total chemerin levels in serum (1:100 dilution) and explant supernatant (1:20 dilution) were determined by ELISA (R&D) as per manufacturer’s instructions. For analysis of chemerin levels in colon explant cultures, results were normalized to the weight of colon tissue that was collected.

Bioactive chemerin levels in serum (1:10 dilution) were determined using the Tango assay for mouse CMKLR1 activation as previously described (Barnea et al. 2008; Ernst et al. 2010; Parlee et al. 2010). Briefly, HTLA cells were transfected with mCMKLR1. After 24 h the cells were treated with the sample of interest for 24 h. Cells were then lysed and luminescence detected as a read-out of beta-arrestin recruitment/CMKLR1 activation. Luciferase activity was normalized to *β*-gal activity as an internal control and the concentration of bioactive chemerin was determined by plotting against a standard curve generated with recombinant mouse chemerin (aa17–156; R&D).

### Western blot analysis

For analysis of chemerin levels in colon explant cultures via SDS-PAGE, 10 ng of recombinant mouse chemerin (aa17–156; R&D) or 30 *μ*L of culture supernatant was boiled with 6× SDS-PAGE loading buffer containing 2-mercaptoethanol. Proteins were separated on a 15% gel and transferred to nitrocellulose membrane (BioRad Laboratories, Mississauga, ON). Blots were blocked with Odyssey blocking buffer (LI-COR, Lincoln, NE) and then incubated with antichemerin (R&D) overnight at 4°C followed by incubation with an IRDye800-conjugated secondary antibody (LI-COR). Immunoreactive bands were detected by scanning at 800 nm using the Odyssey Infrared System (LI-COR Biosciences, Lincoln, NE).

### Statistical analysis

All statistical analysis was performed using GraphPad Prism (version 5.0b; La Jolla, CA). All parametric data are shown as mean ± SEM. For comparisons, a Student’s *t*-test (two groups) or one-way analysis of variance (ANOVA) with a Tukey posttest (3+ groups) was performed, with repeated measures when possible (chemerin levels throughout DSS treatment). A two-way ANOVA with a Bonferonni posttest was used to compare across genotypes and before/after DSS treatment or healthy/DSS-treated animals. Nonparametric data (clinical illness and inflammation scores) are presented as median and were compared using a Kruskal–Wallis test with Dunn’s multiple comparison posttest. Within a particular day of treatment, chemerin versus PBS injection groups were compared using a Mann–Whitney test. For all statistical tests, a significant difference was considered *P *<* *0.05.

## Results

### Chemerin and CMKLR1 expression increase in the cecum and distal colon following DSS treatment

**Figure 1:**
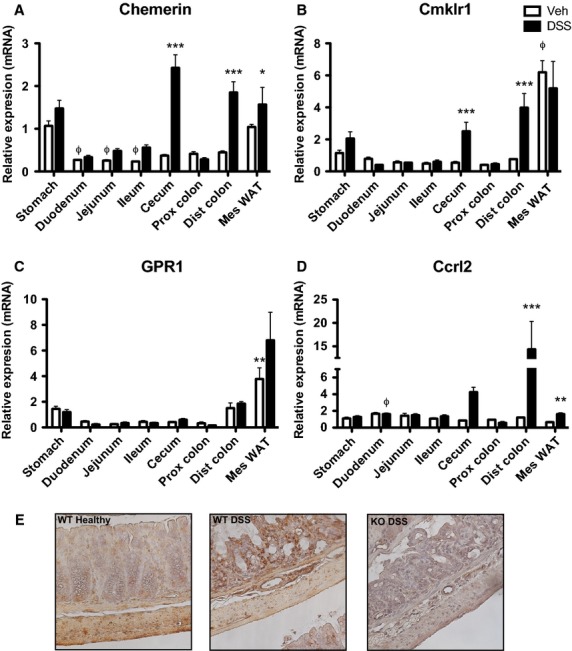
Chemerin and CMKLR1 expression increase in the cecum and distal colon following DSS treatment. RNA from the indicated tissues was isolated at sacrifice from 6- to 8-week-old mice that received normal drinking water (Veh) or 5% DSS treatment (*n *=* *5). qPCR analysis was performed to look at changes in chemerin (A), CMKLR1 (B), GPR1 (C), and CCRL2 (D) expression. CMKLR1 expression throughout the colon of healthy and DSS-treated animals was visualized by immunohistochemistry (E, 40× magnification). Φ Represents *P *<* *0.05 compared to stomach for vehicle-treated animals and * represents *P *<* *0.05, **P <* *0.01, and ***P <* *0.001 for vehicle versus DSS-treated animals.

We first investigated the expression of chemerin and chemerin receptors throughout the mouse GI tract. Quantitative PCR (qPCR) analysis demonstrated that chemerin expression was highest in normal mouse stomach, with significantly lower levels in the duodenum, jejunum, and ileum (Fig.1A). Basal expression of CMKLR1 or CCRL2 was generally similar across the GI tract with the exception of significantly higher levels of CMKLR1 in mesenteric WAT (Fig.1B and D). However, GPR1 expression was modestly decreased in all tissues except for the distal colon and WAT when compared to stomach (Fig.1C). We next examined changes in chemerin and receptor expression within the GI tract following DSS-induced colitis. This model reproducibly induces disease which resembles human UC as it produces a cecum and distal colon-specific mucosal, ulcerating inflammation; however, some features of CD such as transmural inflammation, may also be present (Goyal et al. 2014). Tissues were isolated 1 day following the removal of DSS from the drinking water in order to minimize any confounding effects of residual DSS on our analyses. qPCR analysis revealed a dramatic increase in chemerin expression in the cecum and distal colon (∼6.5-fold) and a modest 1.5-fold increase in mesenteric WAT (Fig.1A). Notably, there was no change in chemerin expression in the proximal colon. Similarly, CMKLR1 expression increased approximately fivefold in both the cecum and distal colon (Fig.1B). There was a modest increase in CMKLR1 expression in the stomach but not in any other tissue examined. While CCRL2 expression increased dramatically in the distal colon and cecum (∼12- and ∼5-fold, respectively; Fig.1D) no changes in GPR1 expression were observed following DSS treatment (Fig.1C).

Chemokine-like receptor 1 is highly expressed on leukocytes such as macrophages, dendritic cells, and natural killer cells, as well as intestinal epithelial cell lines (Wittamer et al. 2003; Zabel et al. 2005; Campbell et al. 2010). In order to investigate whether the increase in CMKLR1 expression in the distal colon was in the cells comprising the colon (e.g., epithelial or muscle cells) or a result of increased cell infiltrate, we performed immunohistochemistry to visualize CMKLR1 expression along the entire length of the colon. An increase in CMKLR1 expression was observed in colons isolated from animals that received DSS treatment compared to healthy animals, and this was largely on infiltrating immune cells that can be observed in the submucosa (Fig.1E). As expected, there was no detection of CMKLR1 in KO animals.

### Chemerin secretion from the colon increases in a proximal–distal gradient

**Figure 2:**
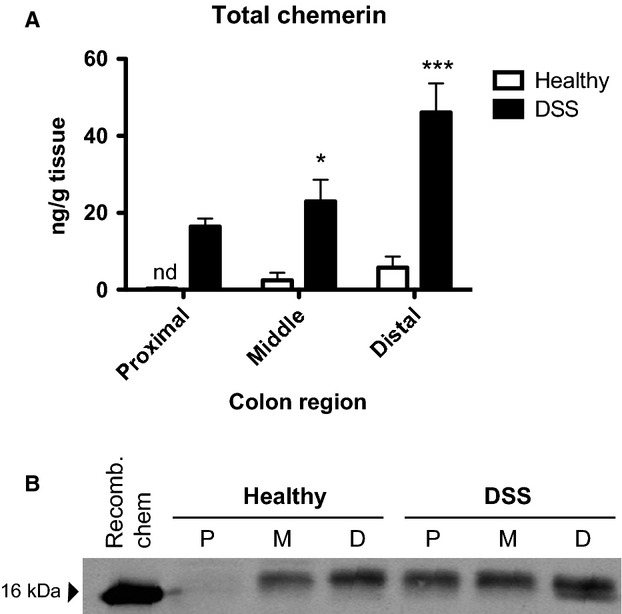
Chemerin secretion increases in a proximal–distal gradient in the colon following DSS treatment. Colons were isolated from 6- to 8-week-old healthy or 5% DSS-treated mice at sacrifice, cut into three segments, and cultured. After 24 h of culture, supernatant from the colon explants was collected and an ELISA performed to determine levels of total secreted chemerin (A). Supernatant from colon explant cultures was also examined via SDS-PAGE with 10 ng of recombinant mouse chemerin (aa17–156, 16 kDa reference) as a control (B). Representative image is shown. *n *=* *6, where *P <* *0.05 and ***P <* *0.001. nd, not detected; P, proximal; M, middle; D, distal.

Having established that chemerin expression increased in the GI tract following DSS treatment, particularly in the distal colon, we next investigated changes in chemerin secretion from the colon. To do this, we generated explant cultures from microdissected regions of the colon from healthy or DSS-treated mice and performed an ELISA to examine total chemerin levels. In healthy animals, chemerin was detected at low levels in the supernatant from middle and distal colon segments but not in the supernatant derived from proximal colon. Consistent with the gene expression data, chemerin expression increased in all three regions following DSS treatment. Specifically, there was a small increase in the proximal segment, a significant increase in the middle segment, and a dramatic increase (from ∼8 to 46 ng/g tissue) in the distal segment (Fig.2A). Similarly, chemerin was undetectable by western blot analysis in the supernatant from proximally derived colon segments, but increased along a proximal–distal gradient in both healthy and DSS-treated animals (Fig.2B). In all segments of the colon, a band larger in size than the recombinant chemerin control (∼16 kDa) was detected in the explant supernatant. This suggests the presence of prochemerin (18 kDa) and indicating a generally absent or very low rate of extracellular processing. However, the detection of an additional band in the distal region from DSS-treated animals which appeared to be similar in size to recombinant chemerin indicates that in addition to increased secretion of chemerin, proteolytic processing of prochemerin occurred selectively in response to DSS-induced inflammation in this colon segment.

### Circulating levels of bioactive:total chemerin increase following DSS treatment

**Figure 3:**
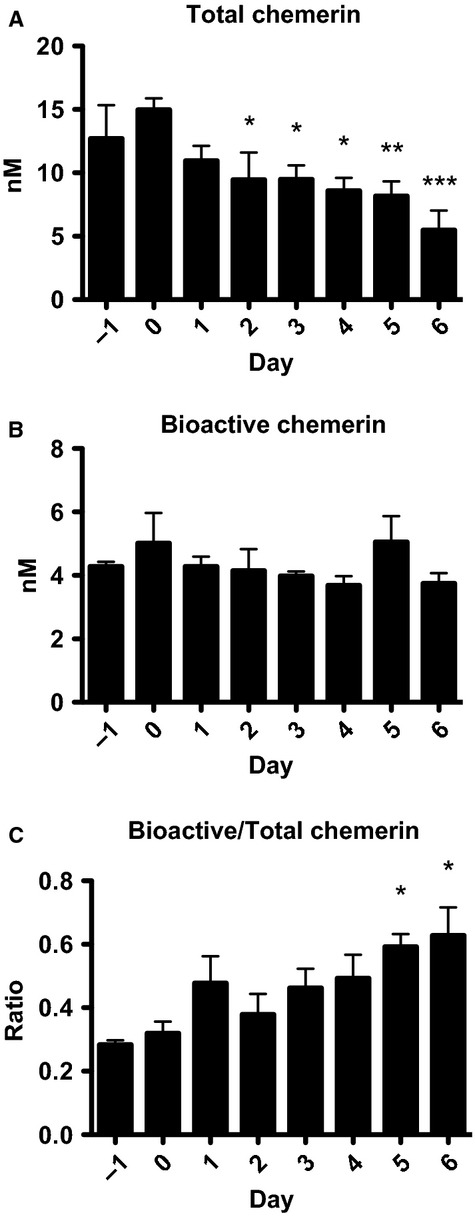
Total circulating chemerin levels decrease, but the ratio of bioactive:total chemerin increases, following DSS treatment. Serum was collected from 6- to 8-week-old mice the day before and on each day of 5% DSS treatment. An ELISA for total chemerin levels (A) and a Tango assay for mCMKLR1 activation (B) were performed. The ratio of bioactive:total chemerin levels was then calculated (C). *n *=* *6, where * represents *P *<* *0.05, **P <* *0.01, and ***P <* *0.001 compared to the day before DSS treatment was initiated.

We next examined whether circulating chemerin levels change throughout the course of DSS treatment. To do this, blood samples were collected from wild-type mice 1 day before starting DSS treatment and every day through to sacrifice. Total chemerin levels as measured by ELISA were decreased starting at day 2 and progressively decreased ∼60%, until sacrifice (Fig.3A). We then investigated chemerin bioactivity in serum using the cell-based Tango assay, which assesses levels of CMKLR1 activation (Barnea et al. 2008). This demonstrated that chemerin bioactivity in serum did not change over the course of the experiment (Fig.3B). Notably, this results in an increase in the ratio of circulating bioactive to total chemerin, which is significantly higher on days 5 and 6 of the study (Fig.3C). Thus, despite a decrease in total chemerin levels, the relative level of bioactive chemerin in circulation was increased following the induction of DSS-induced colitis.

### Signs of DSS-induced illness develop more slowly in CMKLR1-null mice

**Figure 4:**
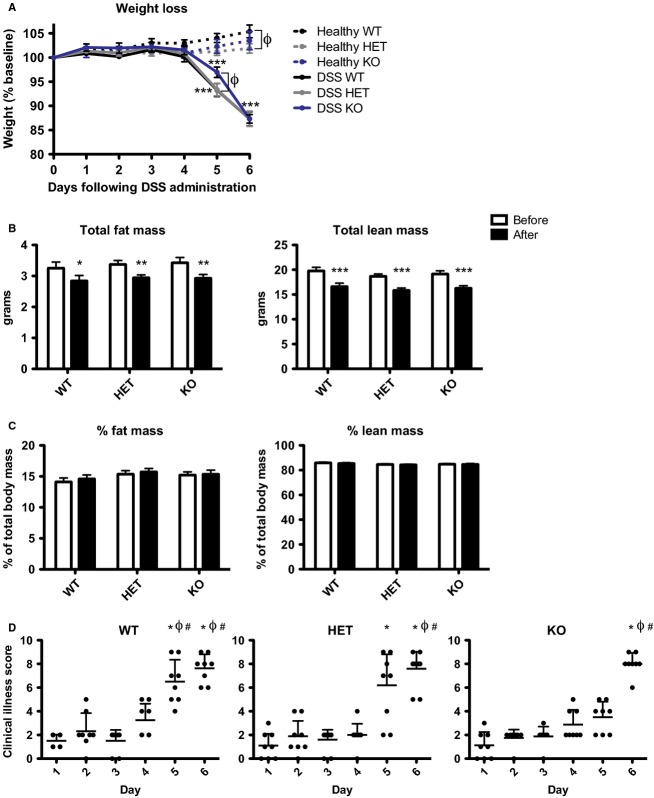
CMKLR1 KO mice have decreased weight loss on day 5 of treatment and develop signs of clinical illness more slowly than WT mice. Body weight of 6- to 8-week-old mice that received normal drinking water (healthy) or 5% DSS was monitored daily and expressed relative to initial weight on day 0 (A). DEXA analysis was performed before (day −1) and after 5% DSS treatment (day 6, before sacrifice) in 6- to 8-week-old animals. The absolute amounts of fat and lean mass (B) were recorded. These values were then normalized to total body weight to calculate percent body composition (C). Mice were evaluated daily for signs of clinical illness and a score was assigned based on weight loss, stool consistency, and the presence or absence of blood in the stool (D). *n *=* *6–9 per genotype, where in (A) *** represents *P *<* *0.001 relative to day 0 and Φ represents *P *<* *0.05 compared to WT within a treatment group on a particular day. In (B) and (C) * represents *P *<* *0.05, **P <* *0.01, and ***P <* *0.001 compared to before DSS treatment. In (D) * represents *P *<* *0.05 compared to day 1, Φ represents *P *<* *0.05 compared to day 2, and # represents *P *<* *0.05 compared to day 3.

With the observation that bioactive chemerin levels increased both locally and systemically following the induction of IBD, and that CMKLR1 was the only signaling chemerin receptor to exhibit parallel changes in expression, we investigated the impact of CMKLR1 loss on colitis. To do this, we performed the DSS-induced colitis model with 6- to 8-week-old CMKLR1 wild-type (WT), heterozygous (HET), or knockout (KO) littermate mice and monitored the development of colitis. There was no difference in body weight between any of the three genotypes at the start of the study (day 0). Healthy control animals that received normal drinking water gained weight over the 6 days of the study, with WT mice gaining more weight (5.4%) than HET mice (1.9%) or KO (3.5%) by day 6 (Fig.4A). Following treatment with DSS, all three genotypes exhibited significant weight loss on days 5 and 6 when compared to day 0. Dual-energy X-ray absorptiometry (DEXA) analysis on day 6 revealed that all genotypes lost a similar amount of lean and fat mass (∼2.8 g and 0.4 g, respectively; Fig.4B), with no difference in percent body composition before versus after DSS treatment (Fig.4C). However, despite weight loss on day 6, KO mice exhibited significantly less weight loss than WT or HET on day 5 of the study (Fig.4A).

Mice were assessed daily for signs of clinical illness and a score based on weight loss, stool consistency, and the presence of blood in the stool was assigned. There was no difference in clinical illness score between genotypes on day 0 or for healthy mice throughout the study (data not shown). By day 6, all genotypes received similar clinical illness scores of ∼8. While KO mice were ultimately not protected from developing signs of clinical illness, the onset of illness occurred more slowly in HET and KO mice (Fig.4D). Importantly, KO mice exhibited a significantly lower clinical illness score (3.5 vs. 7) than WT on day 5.

### Local colonic inflammation is similar between CMKLR1 WT and KO mice

**Figure 5:**
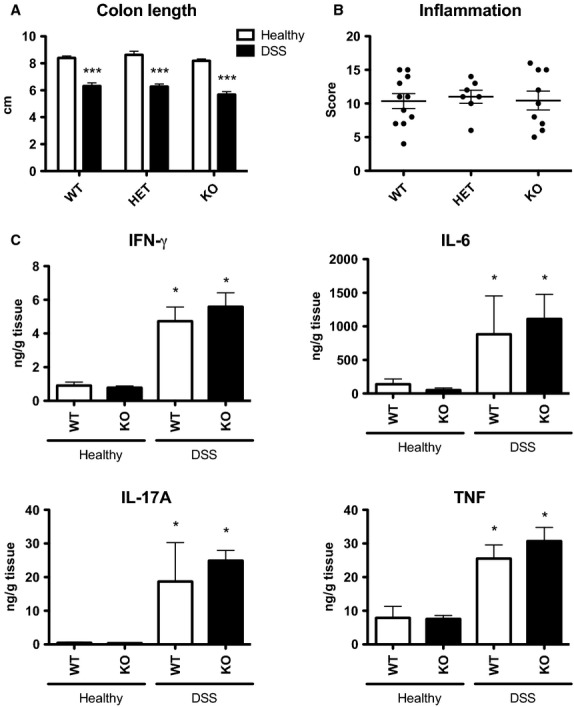
CMKLR1 WT and KO mice have similar levels of local inflammation in the colon following DSS treatment. At sacrifice, the colon was excised from 6- to 8-week-old animals that had received normal drinking water (healthy) or 5% DSS-treatment and measured (A). A blinded investigator scored the level of inflammation in the colon based on the presence of edema, crypt structure, cell infiltration, ulcer formation and size, and hyperplasia, on hematoxylin and eosin stained sections (B). The levels of proinflammatory cytokines IFN-*γ*, IL-6, IL-17A, and TNF secreted from 24-h colon explant cultures isolated at sacrifice was measured using a multiplex assay (C). *n *=* *6–11 per genotype, where * represents *P *<* *0.05 and ***P <* *0.001 compared to healthy animals of the same genotype.

In order to assess colon inflammation among the genotypes following DSS-induced colitis, colons were excised and measured at sacrifice. There was no difference in colon length between genotypes in healthy animals (data not shown). Consistent with the similar clinical illness scores observed by day 6, all three genotypes exhibited similar levels of colon shortening (∼25%) following DSS treatment (Fig.5A). The level of colon inflammation, based on the presence of edema, crypt structure, cell infiltration, ulcer formation and size, and hyperplasia was also assessed. There were no signs of inflammation in healthy mice (data not shown) and no difference in inflammation score between genotype (Fig.5B). As an additional marker of local inflammation, levels of proinflammatory cytokines that are increased following intestinal inflammation (Neurath 2014) were assessed in the supernatant of colon explants isolated from 6- to 8-week-old animals. As predicted, significant increases in interferon (IFN)-*γ*, interleukin (IL)-6, IL-17a, and tumor necrosis factor (TNF) were observed for animals that received DSS treatment compared to healthy controls. However, for both healthy and DSS-treated animals, there were no differences in the levels of these cytokines between WT and KO animals (Fig.5C). Considered altogether, this indicates that local levels of inflammation were similar in WT and KO animals at the conclusion of the treatment period.

### Loss of CMKLR1 alters systemic inflammation response to DSS-induced colitis

**Figure 6:**
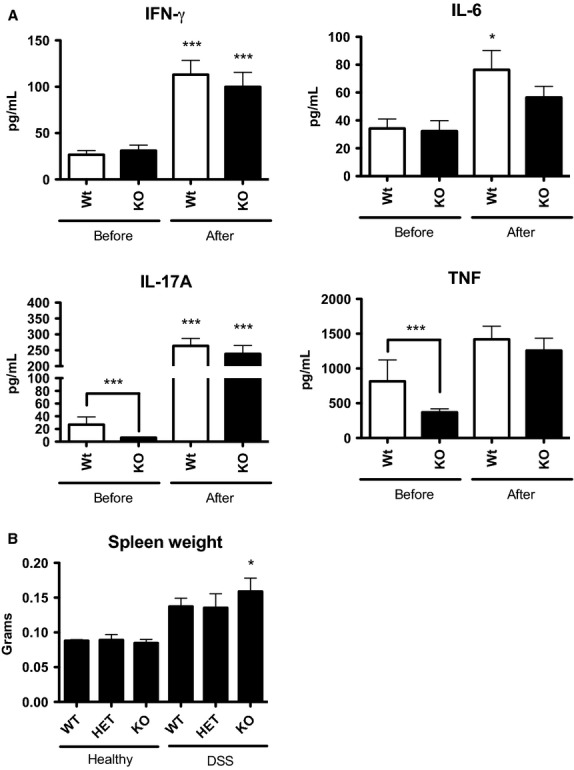
CMKLR1 KO mice have differences in systemic parameters of inflammation. Circulating levels of the proinflammatory cytokines IFN-*γ*, IL-6, IL-17a, and TNF were measured in the serum of 6- to 8-week-old WT or KO mice before and after 5% DSS treatment using a multiplex assay (A). Following DSS treatment, the spleens were excised from mice and weighed (B). *n *=* *8 (A) and *n *=* *6–9 (B) per genotype, where * represents *P *<* *0.05 and ***P <* *0.001 compared to healthy animals of the same genotype or as indicated.

The secretion of proinflammatory cytokines was measured in the serum of 6- to 8-week-old mice before and after DSS treatment in order to assess changes in systemic inflammation (Fig.6A). Levels of IFN-*γ* and IL-6 were similar in WT and KO animals before DSS treatment. Notably, levels of both IL-17a and TNF were decreased in healthy KO animals. Following DSS treatment, significant increases in circulating IFN-*γ*, IL-6, IL-17a, and TNF levels were observed in WT animals. Similarly, increased levels of these cytokines were also observed in KO animals; however, the increase in IL-6 levels was not significant and modest compared to that of WT animals (Fig.6A). As an additional systemic parameter of inflammation, spleen weight was measured at sacrifice. In healthy animals, spleen weight was equivalent across the three genotypes. While a general increase in spleen weight was observed for all genotypes in response to DSS treatment (Fig.6B), this was significant only for KO animals when compared to healthy controls. Thus, the constitutive loss of CMKLR1 altered some systemic inflammatory parameters both basally and in response to DSS-induced colitis.

### Locally secreted chemerin levels are increased in CMKLR1 KO mice

**Figure 7:**
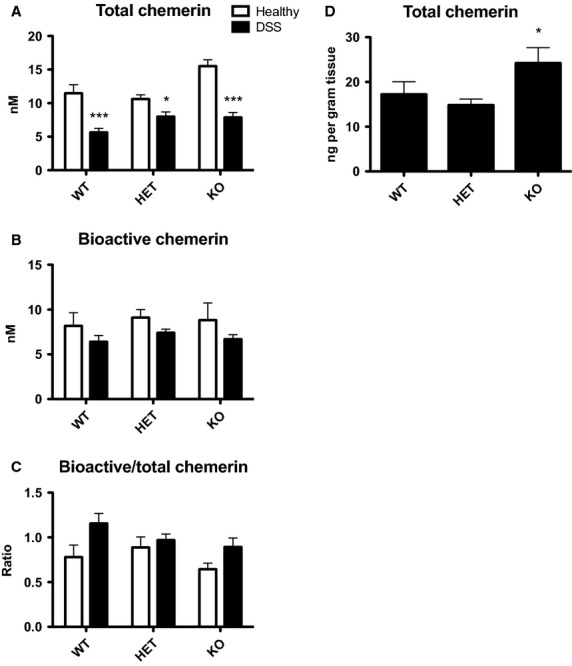
Changes in circulating chemerin levels are similar between genotypes but CMKLR1 KO mice have increased local secretion of chemerin. Serum was collected via cardiac puncture at sacrifice from 6- to 8-week-old mice that received normal drinking water (healthy) or 5% DSS treatment. Total chemerin levels were measured via ELISA (A) and bioactive chemerin levels were measured using the Tango assay for mouse CMKLR1 activation (B). The ratio of bioactive:total chemerin was then determined (C). Total chemerin levels were also measured in the supernatant from colon explant cultures isolated at sacrifice using an ELISA (D). *n *=* *6–9 per genotype, where * represents *P *<* *0.05 and ***P <* *0.001 compared to healthy controls (A) or WT (B).

In order to determine whether there was a difference in circulating chemerin levels between the genotypes following DSS treatment, total serum chemerin levels were evaluated at sacrifice. Consistent with Figure3, all three genotypes exhibited approximately a 50% decrease in total chemerin levels (measured by ELISA) following DSS treatment (Fig.7A). Bioactive chemerin levels (measured by Tango bioassay) were similar in healthy and DSS-treated mice regardless of genotype (Fig.7B) and thus resulted in a trend for increased bioactive:total chemerin ratio for all genotypes. Importantly, there was no significant difference in circulating total or bioactive chemerin levels between genotypes (Fig.7C). However, analysis of total chemerin levels secreted from colon explant cultures revealed a significant increase in local chemerin secretion from colons isolated from KO mice compared to WT or HET (Fig.7D).

### Older CMKLR1 KO mice exhibit signs of clinical illness more slowly, have decreased fat mass, and increased local chemerin levels following DSS treatment

**Figure 8:**
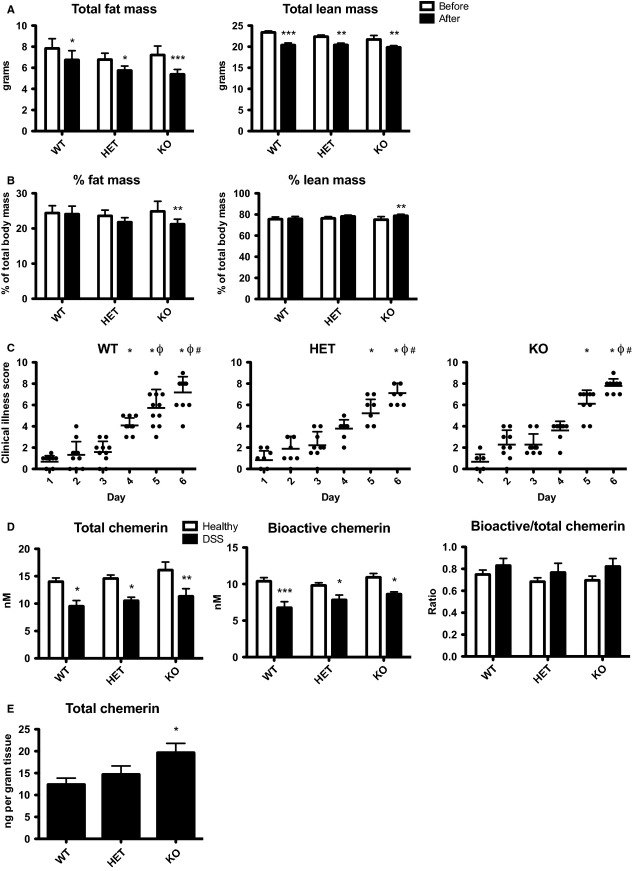
CMKLR1 KO mice (14- to 16-week-old) lose a greater percentage of fat mass, develop signs of clinical illness more slowly, and have increased local levels of chemerin secretion than WT mice. DEXA analysis was performed before (day -1) and after 5% DSS treatment (day 6, before sacrifice) in 14- to 16-week-old mice that receive normal drinking water (healthy) or 5% DSS. The absolute amounts of fat and lean mass (A) were recorded. These values were then normalized to total body weight to calculate percent body composition (B). Mice were evaluated daily for signs of clinical illness and a score was assigned based on weight loss, stool consistency, and the presence or absence of blood in the stool (C). Serum was collected via cardiac puncture at sacrifice and total chemerin levels were measured via ELISA, bioactive chemerin levels were measured using the Tango assay for mouse CMKLR1 activation, and the ratio of bioactive:total chemerin was determined (D). Total chemerin levels were also measured in the supernatant from colon explant cultures isolated at sacrifice using an ELISA (E). *n *=* *6–10 per genotype, where in (A) and (B) * represents *P *<* *0.05, **P <* *0.01, and ***P <* *0.001 compared to before DSS treatment. For (C) * represents *P *<* *0.05 compared to day 1, Φ represents *P *<* *0.05 compared to day 2, and # represents *P *<* *0.05 compared to day 3. In (D) and (E) * represents *P *<* *0.05, **P <* *0.01, and ***P <* *0.001 compared to healthy controls (D) or WT (E).

Previous work has demonstrated that CMKLR1 KO mice exhibit metabolic perturbations at 14 weeks of age that are not present at 6 weeks of age (Ernst et al. 2012). In order to determine whether age was a factor in the development of colitis in CMKLR1 KO mice, we repeated the DSS-induced colitis experiments in an older group of mice (14–16 weeks). In comparison to the study with 6- to 8-week-old mice, healthy animals did not gain weight over the course of the 6-day experiment. However, as with the younger group of mice, all three genotypes lost an equivalent amount of weight on days 5 and 6 compared to day 0 following DSS treatment (data not shown). DEXA analysis on day 6 revealed that while all genotypes lost both lean and fat mass (∼2.5 g and ∼1 g, respectively; Fig.8A), only KO mice exhibited a significant decrease in percent body fat and an increase in percent lean mass compared to before DSS treatment (Fig.8B). Consistent with the pattern in clinical illness for young adult mice, when compared to day 1, clinical illness scores were significant as early as day 4 in older WT mice but not until day 5 and 6 for HET and KO mice. However, all three genotypes had equivalent clinical illness scores by day 6 (Fig.8C). Similar to the younger group of mice, all three genotypes of 14- to 16-week-old mice exhibited similar levels of local inflammation, with ∼25% colon shortening following DSS treatment and an inflammation score of ∼10. However, CMKLR1 KO mice had increased spleen weight following DSS treatment (data not shown). Therefore, as with 6- to 8-week-old mice, older KO mice developed clinical illness more slowly, although ultimately reaching a similar level of illness and local colon inflammation as WT mice.

Compared to 6- to 8-week-old animals, 14- to 16-week-old mice had higher levels of total circulating chemerin (15 nmol/L compared to 10 nmol/L). Similar to the younger group, older mice exhibited a decrease in total chemerin levels following DSS treatment. However, unlike the younger group, a significant decrease in bioactive levels was also observed. The resulting effect was a more modest increase in bioactive:total chemerin ratio compared to 6- to 8-week-old animals (Fig.8D). There was no difference between genotypes with relevance to circulating chemerin levels. However, there was significantly higher local chemerin secretion from colon explant cultures isolated from 14- to 16-week-old KO mice compared to WT or HET (Fig.8E). Thus, for both age groups, while there is no difference in circulating chemerin levels between genotype, locally secreted chemerin levels are increased in KO animals.

### Bioactive chemerin injection does not influence the severity of DSS-induced colitis

**Figure 9:**
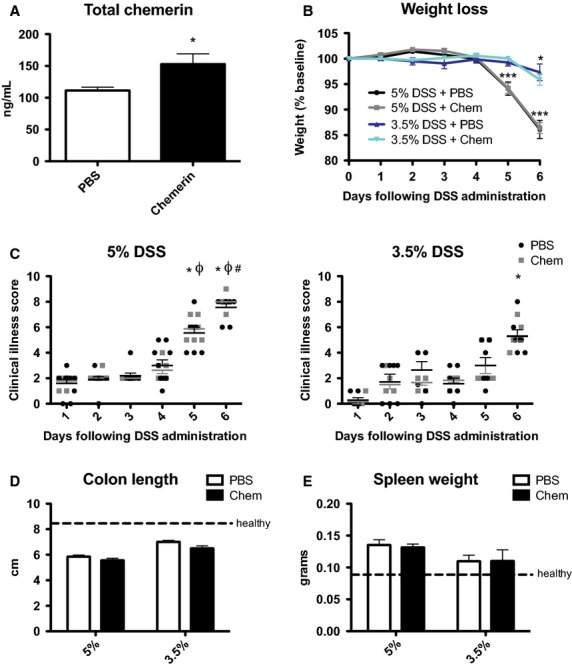
Increased levels of circulating active chemerin do not affect DSS-induced colitis. Recombinant chemerin (aa17–156) or PBS (control) was administered to 6- to 8-week-old WT animals via intraperitoneal injection every other day. Total chemerin levels in circulation 48 h following injection were measured using an ELISA (A). Body weight was monitored daily for animals receiving 3.5% or 5% DSS and expressed relative to initial starting weight (A). Clinical illness was also scored daily for animals receiving 5% (C) or 3.5% (D) DSS. At sacrifice, the colon was excised and measured (D) and spleen weight recorded (E). *n *=* *6–9 per group, where in (A) * represents *P *<* *0.05, in (B) * represents *P *<* *0.05 and ***P <* *0.001 compared to day 0, and in (C) * represents *P *<* *0.05 compared to day 1, Φ represents *P *<* *0.05 compared to day 3, and # represents *P *<* *0.05 compared to day 3.

To determine the impact of increased circulating bioactive chemerin levels on the development and/or progression of DSS-induced colitis, we injected 6- to 8-week-old WT mice with bioactive chemerin (aa17–156) or PBS control every other day throughout DSS treatment starting on day 0. Intraperitoneal injection of 500 ng chemerin increased circulating levels by 30% (Fig.9A). This increase in circulating chemerin is physiologically relevant as it is similar to the increase in bioactive:total chemerin ratio in mice administered DSS compared to healthy control (Fig.7C).

As expected, mice exhibited significant weight loss and had increased clinical illness scores on days 5 and 6 of DSS treatment; however, there was no difference between PBS control and chemerin-injected animals (Fig.9B and C). Consistent with this, both PBS and chemerin-injected animals exhibited similar levels of colon shortening and increases in spleen weight at sacrifice on day 6 (Fig.9D and E). To confirm that chemerin-injected animals were not inhibited from developing more severe colitis than control animals because of relatively high levels of inflammation on the 5% DSS treatment regime, we decreased the amount of DSS in drinking water to 3.5%. This resulted in reduced amounts of weight loss, clinical illness, colon shortening, and spleen weight increase (Fig.9B–E). However, there was no difference in any of these parameters between PBS and chemerin-injected animals, demonstrating that increased bioactive circulating chemerin levels do not influence the development of DSS-induced colitis.

## Discussion

While the etiology of IBD is largely unknown, it is generally believed that genetic, environmental, and microbial factors combine to trigger inappropriate immune responses that involve overproduction of different proinflammatory mediators (Hanauer 2006; Kaser et al. 2010; Neurath 2014). These cytokines and chemokines play a crucial role in controlling intestinal inflammation. Our results support a role for chemerin in the complex signaling network that underlies the pathogenesis of this inflammation. In the current study, we demonstrate that chemerin expression, secretion, and bioactivity are highest in regions of the colon that are the most inflamed. While the cellular source of chemerin was not directly investigated in this study, previous studies have demonstrated that chemerin is expressed in both fetal and cultured adult epithelial cells (Maheshwari et al. 2009). We proposed that chemerin is secreted from the inflamed colon and functions as a chemoattractant to recruit CMKLR1-expressing cells to sites of tissue damage. Surprisingly, while loss of CMKLR1 expression delayed the progression of DSS-induced colitis, it did not ultimately confer protection against disease severity.

A key finding from this study is that local chemerin levels appear to be more biologically relevant in the development of IBD than systemic chemerin levels. We demonstrate that the increase in chemerin expression was most dramatic in inflamed tissues in the GI tract (cecum and colon) and that the increase in chemerin secretion correlated with the severity of inflammation in the colon. This is consistent with human data showing that chemerin expression is higher in colon biopsies from UC patients with more severe inflammation, suggesting that local chemerin levels are tightly associated with local colon inflammation (Lin et al. 2014). In contrast, systemic levels of chemerin in UC and CD patients are not clearly related to disease activity in both sexes (Weigert et al. 2010; Waluga et al. 2014). Similarly, in our study neither the DSS-induced decrease in circulating total chemerin for all mice nor the overall higher circulating chemerin in older versus younger mice appeared to have any impact on the disease progression or severity. Furthermore, injection of bioactive chemerin had no observable effect on disease severity. These results are consistent with a study demonstrating that chemerin levels in synovial fluid from arthritic joints are positively associated with disease severity while there was no difference between serum chemerin levels in healthy controls and osteoarthritis patients (Huang et al. 2012). This suggests that local chemerin levels in diseases involving chronic inflammation are a more important determinant of disease activity than systemic levels.

Importantly, this is the only published study to examine levels of chemerin bioactivity in a model of IBD. We report that despite a decrease in total circulating chemerin levels, the ratio of bioactive:total chemerin is increased in circulation with colitis. Furthermore, the presence of an extra band via SDS-PAGE analysis in the most inflamed region of the colon suggests that local processing of chemerin may occur in DSS-induced inflammation. Thus, bioactive chemerin represents a novel biomarker that reflects the extent of inflammation. Although the source of chemerin bioactivation is unknown, this could occur via the secretion of chemerin-activating proteases from the colon (e.g., epithelial cells). However, as many enzymes that are known to process prochemerin, such as immune cell-secreted serine and cysteine proteases, are involved in the inflammatory response, it is likely that infiltrating immune cells contribute significantly to the bioactivation of chemerin in the inflamed colon (Rourke et al. 2013). Recently, it was shown that inhibition of serine proteases reduced inflammation induced by DSS administration (Bermudez-Humaran et al. 2015) and therefore targeting enzymes that generate bioactive chemerin may represent a novel method to manipulate endogenous chemerin signaling. As bioactive levels are often overlooked in both clinical and experimental studies, the findings of this study highlight the importance of studying chemerin bioactivity in pathophysiology.

Bioactive chemerin is a potent chemoattractant for cells that express CMKLR1 and the current study demonstrated that the increase in CMKLR1 expression in DSS-induced colitis resulted at least in part from increased cell infiltration into the colon. We hypothesized that mice lacking CMKLR1 would be protected from inflammatory damage and illness associated with DSS-induced colitis. Surprisingly, CMKLR1 KO mice had similar levels of inflammation to wild-type mice at the final time point, although CMKLR1 KO mice did have a delayed onset of weight loss and clinical illness. This could result from a slower infiltration of immune cells into the colon in the absence of CMKLR1. However, it is likely that infiltrating leukocytes are ultimately attracted by other and possibly multiple chemoattractants; for example, inhibition of the neutrophil chemokine receptor, CXCR2, in mice does not absolutely block neutrophil infiltration in this DSS model (Farooq et al. 2009). Similarly, while chemerin contributes to the recruitment of macrophages in the developing fetal intestine, chemerin levels are lower in the mature intestine, indicating that postnatal attraction is initiated by other chemokines (Maheshwari et al. 2009). Future studies that determine the cell type(s) expressing CMKLR1 in the colon following DSS treatment and examination of potential differences in cell infiltration in CMKLR1 KO mice earlier in the study, or whether there are differences in the populations of infiltrating cells in the absence of CMKLR1 could provide insight into the role of chemerin in immune cell recruitment to the inflamed colon. Recently, Lin et al. (2014) did not observe any changes in cell infiltration to the colon following intraperitoneal chemerin injection. However, given the importance of local chemerin levels, a more accurate model to study the effect of chemerin on cell infiltration to the colon would be via local intrarectal administration or tissue-selective forced overexpression of chemerin versus systemic injection. Lin et al. (2014) proposed that chemerin influences IBD pathogenesis by inhibiting M2 macrophage polarization, which is involved in the resolution of IBD. CMKLR1 has been shown to be functional on M1 but not M2 macrophages and it would be interesting to assess leukocyte content in the spleen to examine whether a defect in M1/M2 macrophage polarization or other cell populations might explain the increased spleen size in CMKLR1 KO mice (Herova et al. 2015).

Lin et al. (2014) also reported that injection of 500 ng of chemerin every other day exacerbated the severity of DSS-induced colitis using an 8-day protocol in which mice received DSS in the drinking water for only the first 5 days. While a limitation of DSS-induced colitis models is often variability in the level of inflammation between groups using similar DSS models, in our experience, this resembles a model that examines the effect of chemerin in recovery from acute colitis rather than the induction of colitis. In the current study, we did not observe a difference in the induction of colitis following the injection of bioactive chemerin using the same dosage and timing of injections in similarly aged mice on the same genetic background. Thus, it is possible that while chemerin does not play a significant role in the initiation of colitis, it is important for the resolution of inflammation. Previous studies with CMKLR1 KO mice have suggested both pathogenic (Graham et al. 2009; Demoor et al. 2011) and protective (Cash et al. 2008; Luangsay et al. 2009; Bondue et al. 2011) roles for chemerin signaling in the initiation and resolution of inflammation, respectively. Thus, the role of chemerin in IBD is likely context-specific and future studies examining role of chemerin/CMKLR1 signaling in other DSS models (acute, chronic, recovery) will be useful. Additionally, DSS is a model of chemically induced mucosal injury and has some limitations as a model of IBD. Therefore, the use of other IBD models (TNBS, IL-10 KO, infectious *Citrobacter*) may provide further insight into the role of chemerin signaling in IBD as well as other inflammatory diseases.

Interestingly, our study demonstrates that total circulating levels decrease throughout the course of DSS treatment. In the current study, mice lost twice as much body weight compared to a previous report (Lin et al. 2014) and DEXA analysis revealed a significant loss in fat mass. As WAT is an important modifiable source of circulating chemerin, this weight loss could explain a decrease in circulating levels. Consistent with this, the older group of mice had decreased bioactive chemerin levels that correlated with a dramatic loss of fat mass. Importantly, chemerin expression increased in mesenteric WAT following DSS treatment, suggesting an additional potential source of local adipose-derived chemerin levels during DSS-induced inflammation. A relationship between obesity, mesenteric adiposity, and IBD has been proposed and high-fat diet-induced obesity has been shown to aggravate the pathology of IBD (Teixeira et al. 2011; Moran et al. 2013). CMKLR1 has previously been reported to affect adiposity and thus chemerin/CMKLR1 signaling may represent a novel link between adiposity and IBD (Ernst et al. 2012). However, it is important to note that normalizing total serum chemerin levels by fat mass did not alter the magnitude of decrease in chemerin levels following DSS treatment (data not shown). Importantly, previous studies have shown that fasting significantly decreases serum chemerin levels (Chamberland et al. 2013). As DSS-treated mice eat less food when ill, the decrease in chemerin levels may be an indirect consequence of DSS treatment on feeding. Alternatively, the impact of DSS administration on other sources of circulating chemerin, such as the liver, is unknown. Future studies that investigate how both local and systemic inflammation influence circulating chemerin levels will be important in furthering our understanding of chemerin function in IBD as well as other inflammatory diseases.

The phenotype achieved following DSS-induced colitis in CMKLR1 KO mice was relatively modest and it is important to note that this may be a result of constitutive loss of CMKLR1. Compensatory mechanisms may have developed in these mice and studies with acute modulation of CMKLR1 activation via administration of CMKLR1 inhibitors or conditional GI knockout models will be useful in future studies. Furthermore, upregulation of chemerin secretion from the colon was observed in KO animals and it is possible that chemerin is acting through receptors other than CMKLR1. We were limited by a lack of specific GPR1 and CCRL2 antibodies that function in immunohistochemistry to examine changes in the protein level of these receptors. However, gene expression data demonstrated that while GPR1 expression did not change following DSS treatment, GPR1 expression was relatively high in the distal colon constitutively compared to other mammalian tissues (data not shown; Rourke et al. 2014). Alternatively, CCRL2, which is believed to be a nonsignaling receptor, increased in the same tissues as chemerin and CMKLR1. CCRL2 has been hypothesized to bind chemerin and present to nearby cells, and has been shown to enhance optimal swelling in IgE-mediated anaphylaxis (Zabel et al. 2008). Thus, it is possible that chemerin plays a role in IBD through any combination of CMKLR1, GPR1, and/or CCRL2 signaling and future studies with additional transgenic chemerin and receptor models will be invaluable. Importantly, while we did not directly test the impact of Resolvin E1, the results of this study suggest that CMKLR1 does not mediate the anti-inflammatory effects of Resolvin E1 in the progression of IBD as previously suggested by others (Arita et al. 2005; Ishida et al. 2010).

In summary, this study shows that local chemerin levels and bioactivity are positively associated with the development of colitis and that chemerin represents a novel biomarker reflecting the severity of IBD. Surprisingly, constitutive loss of CMKLR1 is not sufficient to prevent the induction of colitis although CMKLR1 KO mice develop illness more slowly. In contrast with a previous report (Lin et al. 2014), increased systemic chemerin levels do not appear to influence the severity of experimental colitis, suggesting that local chemerin levels play a more important role in the pathogenesis of IBD. Future studies that investigate the role of other chemerin receptors and mechanisms of endogenous chemerin signaling in IBD both more acutely and locally will be important in elucidating the potential of chemerin as a therapeutic target for the treatment of IBD.
